# Application of SVR-Mediated GWAS for Identification of Durable Genetic Regions Associated with Soybean Seed Quality Traits

**DOI:** 10.3390/plants12142659

**Published:** 2023-07-16

**Authors:** Mohsen Yoosefzadeh-Najafabadi, Sepideh Torabi, Dan Tulpan, Istvan Rajcan, Milad Eskandari

**Affiliations:** 1 Department of Plant Agriculture, University of Guelph, Guelph, ON N1G 2W1, Canada; myoosefz@uoguelph.ca (M.Y.-N.); storabi@uoguelph.ca (S.T.); irajcan@uoguelph.ca (I.R.); 2 Department of Animal Biosciences, University of Guelph, Guelph, ON N1G 2W1, Canada; dtulpan@uoguelph.ca

**Keywords:** data-driven, FarmCPU, genome-wide association study, soybean oil, soybean protein, support vector regression

## Abstract

Soybean (*Glycine max* L.) is an important food-grade strategic crop worldwide because of its high seed protein and oil contents. Due to the negative correlation between seed protein and oil percentage, there is a dire need to detect reliable quantitative trait loci (QTL) underlying these traits in order to be used in marker-assisted selection (MAS) programs. Genome-wide association study (GWAS) is one of the most common genetic approaches that is regularly used for detecting QTL associated with quantitative traits. However, the current approaches are mainly focused on estimating the main effects of QTL, and, therefore, a substantial statistical improvement in GWAS is required to detect associated QTL considering their interactions with other QTL as well. This study aimed to compare the support vector regression (SVR) algorithm as a common machine learning method to fixed and random model circulating probability unification (FarmCPU), a common conventional GWAS method in detecting relevant QTL associated with soybean seed quality traits such as protein, oil, and 100-seed weight using 227 soybean genotypes. The results showed a significant negative correlation between soybean seed protein and oil concentrations, with heritability values of 0.69 and 0.67, respectively. In addition, SVR-mediated GWAS was able to identify more relevant QTL underlying the target traits than the FarmCPU method. Our findings demonstrate the potential use of machine learning algorithms in GWAS to detect durable QTL associated with soybean seed quality traits suitable for genomic-based breeding approaches. This study provides new insights into improving the accuracy and efficiency of GWAS and highlights the significance of using advanced computational methods in crop breeding research.

## 1. Introduction

Soybean is one of the most important dual-use leguminous crops and is the main source of protein (~40%) and oil (~20%) for food [1]. Soybean is also an important source of healthy plant-based food products in the human diet due mainly to its nutritional and pharmaceutical properties [2]. Developing soybean cultivars with high oil and protein concentrations has always been one of the major goals of soybean breeding programs [3]. However, these two traits are quantitative traits that are controlled by many minor and major genes and are highly affected by environments [4,5]. Previous studies verified the strong negative correlation between soybean oil and protein and recommended identifying quantitative trait loci (QTL) that might inversely affect those traits [6,7]. Therefore, a deep understanding of the genetic structure of soybean oil and protein concentration would be pivotal in designing efficient molecular breeding approaches [8,9].

Current remarkable progress in high throughput genotyping techniques has provided breeders and geneticists with a unique opportunity to have access to thousands of single nucleotide polymorphisms (SNPs) in a time and cost-effective manner [10,11]. One of the most recommended genetic tools that has been frequently used by breeders and geneticists to detect marker-trait associations (MTAs) for the trait of interest is the genome-wide association study (GWAS) [12,13]. In the last ten years, a variety of statistical methods have been created and applied to speed up computational analyses, enhance the accuracy and statistical powers in GWAS by testing multiple hypotheses across an entire genome [14]. Two of the commonly used GWAS methods are the mixed linear model (MLM) and fixed and random model circulating probability unification (FarmCPU) [14,15]. In addition, several techniques have been suggested to determine genome-wide significance levels and thresholds, such as the Bonferroni correction and false discovery rate (FDR), in order to decrease the occurrence of erroneous discoveries [15,16]. The application of GWAS was widely studied in different plant species, such as wheat [17], maize [18], soybean [19], and sorghum [20], and the primary objective of all these studies was to accelerate the breeding processes through using GWAS-derived molecular markers for the indirect selection of superior genotypes with improved phenotypic values. However, these studies demonstrated that the effectiveness of GWAS in identifying genetic markers linked to quantitative traits depended on the careful selection of GWAS methods and precise experimental conditions [21].

With the availability of affordable next-generation technologies, researchers are now able to capture much of the genetic variation in a given genome and generate large numbers of genomic sequences and genetic properties even in large plant populations [22,23]. The abundance of plant genetic sequences can be categorized as big data due to their compliance with the three Vs, which are volume, velocity, and variety [24,25]. Efficient analysis of large datasets significantly depends upon multiple processes involved in data collection, data processing, and different management challenges identified in the context of big data [26,27]. Therefore, dealing with big datasets, such as high-density SNPs in GWAS, requires intensive computation and the use of modern statistical approaches, such as artificial intelligence (AI) algorithms [28]. Machine learning (ML) is a subset of AI that can be defined as the development of mathematical models that can learn, educate, and make decisions using available datasets [29,30]. The application of ML algorithms can be considered as an alternative approach to current conventional statistical procedures for analyzing SNP markers in a data-driven manner. One of the important ML algorithms is support vector machines (SVM), developed by Vapnik [31], which is based on finding the optimum hyperplane in the number of variables that classify data points within a dataset [32]. Support vector regression (SVR) as a subset of SVM is widely used to solve regression problems [32]. The successful use of the SVR method was reported in phenomics [33], genomics [34], plant tissue culture [35,36], and metabolomics [37]. The use of SVR in GWAS was introduced by de Oliveira et al. [38] in animal science. They tested the efficiency of SVR-mediated GWAS for selecting the most relevant MTAs using the Pearson universal kernel as a fitness function [38]. However, the use of the SVR-mediated GWAS is less studied in plant areas and requires more investigations.

This study aimed to (1) investigate the genetic structure of soybean seed composition traits; (2) conduct a comparative analysis between FarmCPU, a well-known conventional GWAS method, and SVR-mediated GWAS for detecting genomic regions associated with soybean seed composition traits; and (3) identify genes and QTL co-localized with the detected MTAs for soybean seed composition traits. The identified MTAs in this study can be used in different soybean breeding programs for selecting value-added genotypes through the simultaneous selection of all the target seed quality traits at early growth stages.

## 2. Results

### 2.1. Phenotyping Evaluation

**Figure 1:**
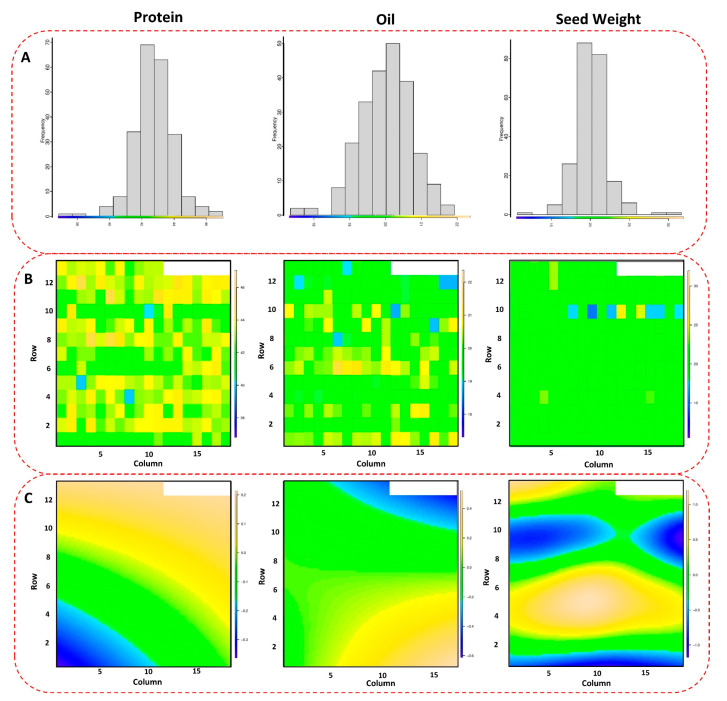
Spatial distribution (**A**) of the tested GWAS panel across four different environments. The scatter plot (**B**) shows the observed values of each soybean genotype, and the color-coded map (**C**) represents the spatial variations in the performance of the genotypes across the environments.

The phenotypic evaluations and collecting process of the soybean yield for the tested panel are explained in detail in [21]. After adjusting the phenotypic plots for each genotype based on spatial analysis, the tested GWAS panel showed significant variations for seed protein, oil, and 100-seed weight across four tested environments (Figure 1A–C). The 100-seed weight had the highest spatial variation in the field, followed by seed protein and seed oil (Figure 1C). The maximum and minimum values for 100-seed weight were 36.49 g and 7.61 g, respectively, with an average of 18.68 g. Seed protein had an average of 39.90% in the tested GWAS panel with maximum and minimum values of 48.77% and 14.51%, respectively. Soybean seed oil also had the maximum and minimum values of 23.37% and 16.61% in the tested GWAS panel, respectively, with an average of 20.03%. Among all the tested traits, seed protein had the highest heritability, with an estimated value of 0.69, followed by seed oil and 100-seed weight with values of 0.67 and 0.60, respectively.

**Figure 2:**
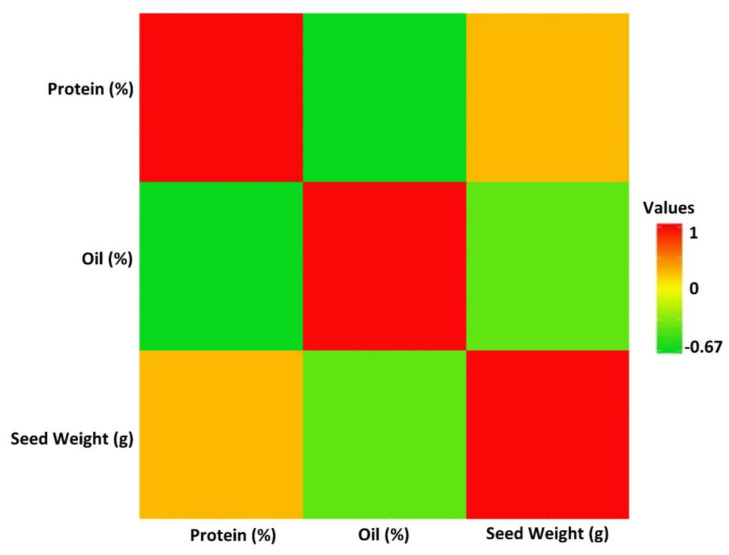
The linear Pearson correlation coefficients between soybean seed protein, oil, 100-seed weight, and yield in the tested GWAS panel across four tested environments. The intensity of the colors represents the strength of the correlations, with red indicating a strong positive correlation.

The linear correlation coefficients (r) estimated among yield and the target seed traits revealed a significant negative correlation (−0.67) between seed protein and oil concentrations (Figure 2). In addition, 100-seed weight was negatively correlated with seed oil, with a value of r = −0.33 (Figure 2). While seed yield had positive correlations with 100-seed weight (r = 0.69) and protein concentration (r = 0.15), it showed a negative correlation with oil concentration (r = −0.10). However, the correlations between seed yield and protein as well as seed yield and oil concentration were not significant (α = 0.05).

#### 2.1.1. Genotyping

Out of 250 soybean genotypes, 23 of them were eliminated because of the high level of missing data, and a total of 17,958 high-quality SNPs were archived from a total of 40,712 SNPs from 227 soybean genotypes and mapped on 20 soybean chromosomes. For the tested association panel, pairwise linkage disequilibrium (LD) between SNPs was calculated based on the correlation coefficient (R^2^) of alleles using 17,958 high-quality SNPs. The minimum number of SNPs (403) was found on chromosome 11, and the maximum number of SNPs (1780) was found on chromosome 18. The average number of SNPs across all the 20 soybean chromosomes was 898, with a mean density of 0.12 cM for every single SNP across the genome.

#### 2.1.2. Population Structure and Kinship

**Figure 3:**
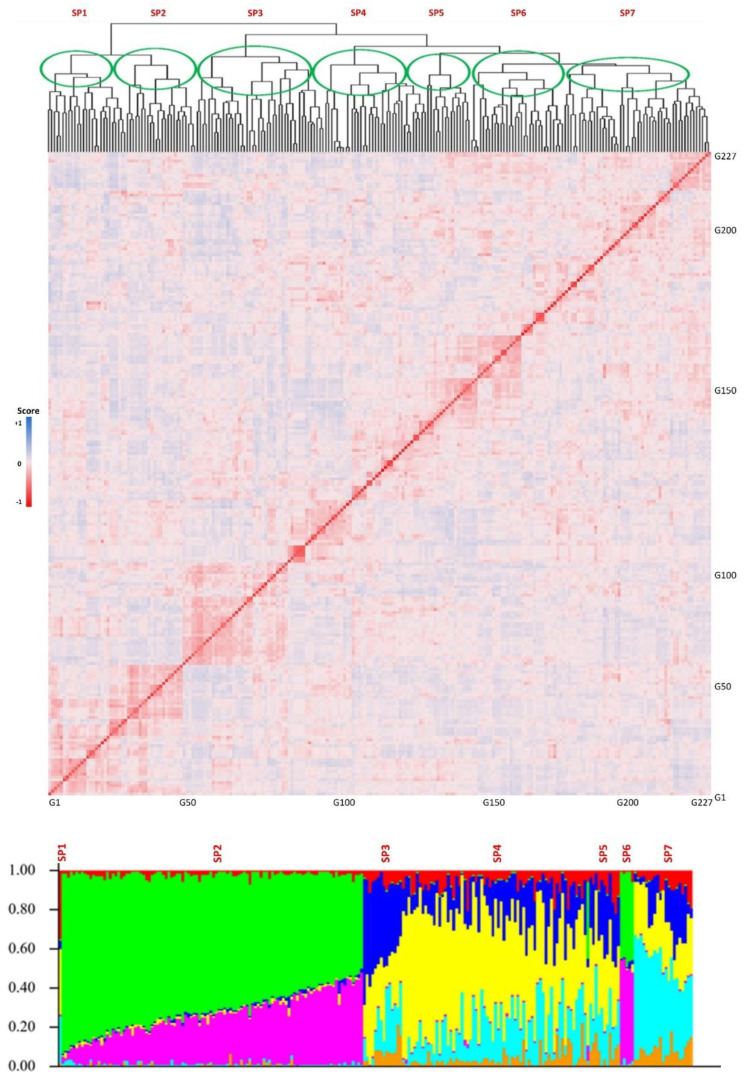
Kinship (**top**) and structure (**bottom**) plots for the tested GWAS panel. The *x*-axis is the number of genotypes used in this GWAS panel, and the *y*-axis is the membership of each subgroup. SP1-SP7 stands for the seven subpopulations.

The genotypic evaluations conducted on the tested GWAS panel provided insights into the population structure, revealing the presence of multiple subpopulations. The results indicated the existence of four to seven distinct subpopulations within the panel. In order to further analyze and consider the population structure as one of the potential cofactors in GWAS analyses, a value of K = 7 was selected as the most appropriate parameter. The population structure analysis, represented in Figure 3, allows for a visual representation of the subpopulations and their distribution within the GWAS panel.

#### 2.1.3. GWAS Analysis

**Figure 4:**
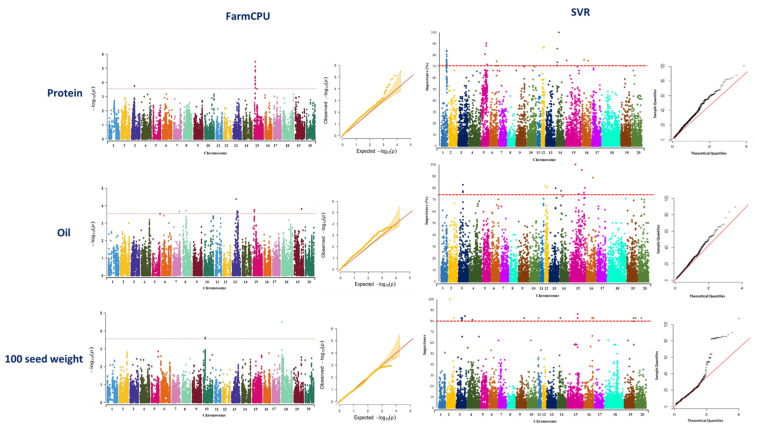
Manhattan and quantile–quantile plots showcasing the results of GWAS studies on soybean seed protein, oil, and 100-seed weight. The plots were generated using two different methods: FarmCPU and SVR. The plots are arranged in a left-to-right sequence, with FarmCPU results displayed first, followed by SVR results.

GWAS analysis using the FarmCPU method identified 15 associated SNP markers for seed protein located on chromosomes 3 and 15 (Figure 4, Table S1). Using SVR-mediated GWAS, a total of 27 SNP markers located on chromosomes 1, 5, 6, 12, 14, 15, and 16 were identified to be associated with soybean protein (Figure 4, Table S2). Genomic regions of chromosome 15 were found to be associated with seed protein using both GWAS methods (Figure 4). In the FarmCPU method, the identified MTA on chromosome 15 was co-localized with previously reported QTL for seed protein (Table 1). In SVR-mediated GWAS, detected MTAs on chromosomes 5 and 16 were co-localized with previously reported QTL for seed protein (Table 1).

A total of 12 SNP markers located on chromosomes 7, 8, 13, 15, and 19 were identified to be associated with seed oil using FarmCPU (Figure 4, Table S3), while using SVR-mediated GWAS, 13 SNP markers located on chromosomes 3, 12, 13, 14, 15, and 16 were found to be associated with this trait (Figure 4, Table S4). Chromosome 15 was the only chromosome in which some of the MTAs were found associated with the trait using both GWAS methods (Figure 4). Most of the detected MTAs by SVR-mediated GWAS were co-localized with six previously reported oil-related QTL such as seed long-chain fatty acid and seed stearic (Table 2). However, most of the detected MTAs by FarmCPU were co-localized with QTL related to leaf carotenoid content, soybean cyst nematode, seed protein, water use efficiency, and soybean sudden death syndrome (Table 2).

For 100-seed weight, totals of 3 and 22 SNP markers were identified underlying the trait using FarmCPU and SVR-mediated GWAS methods, respectively (Figure 4). Detected SNP markers using FarmCPU were located on chromosomes 10 and 18 (Figure 4, Table S5), whereas identified SNP markers using SVR-mediated GWAS were located on chromosomes 2, 3, 4, 9, 11, 14, 15, 16, 19, and 20 (Figure 4, Table S6). Most of the detected MTAs using SVR-mediated GWAS were co-localized with previously reported QTL related to the first flower formation, number of nodes, plant height, soybean cyst nematode, water use efficiency, and maturity date (Table 3). Most of the detected MTAs using the FarmCPU method were co-localized with previously reported QTL related to soybean cyst nematode and water use efficiency (Table 3). Most of the detected chromosomes using SVR-mediated GWAS were similarly detected for yield in the previous study [58].

#### 2.1.4. Extracting Candidate Genes Undelaying Detected QTLs

**Figure 5:**
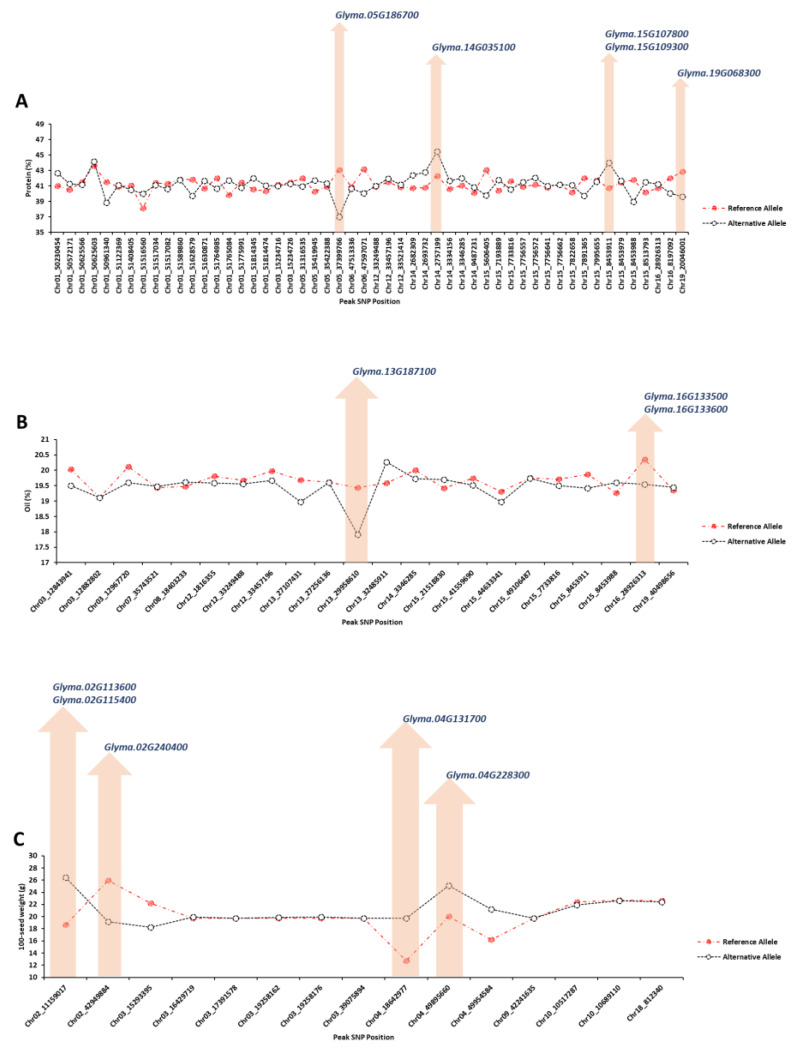
GWAS results of the top significant SNPs associated with soybean seed protein (**A**), oil (**B**), and 100-seed weight (**C**) across different environments. The *y*-axis represents the value of the trait of interest, and the *x*-axis represents the genomic position of each SNP on the soybean genome.

Considering the 150 kbp upstream and downstream flanking regions for each peak SNP with high allelic effect, the potential candidate genes were identified using gene annotation, previous studies, and enrichment tools. For seed protein concentration, four peak SNPs (Chr05_37399766, Chr14_2757199, Chr15_8453911, and Chr19_20046001) had the highest allelic effect compared to other identified peak SNPs (Figure 5A). Five candidate genes, *Glyma.05G186700* (GO:0006865), *Glyma.14G035100* (GO:0009888), *Glyma.15G107800* (GO:0016926), *Glyma.15G109300* (GO:0009658), and *Glyma.19G068300* (GO:0010099), were detected as the strong candidate genes governing seed protein, which encode amino acid transport, tissue development, protein desumoylation, chloroplast organization, and regulation of photomorphogenesis, respectively (Figure 5). For soybean seed oil, two peak SNPs, Chr13_29958610 and Chr16_28926313, had the highest allelic effect compared to other detected peak SNPs (Figure 5B). Based on the gene annotation and expression within QTL, *Glyma.13G187100* (GO:0008168), *Glyma.16G133500* (GO:0009697), and *Glyma.16G133600* (GO:0016887) were identified as the strong candidate genes underlying soybean seed oil, which encode methyltransferase activity, salicylic acid biosynthetic process, and ATPase activity, respectively (Figure 5B). The peak SNPs of Chr02_11159017, Chr02_42949884, Chr04_18642977, and Chr04_49895660 had the highest allelic effect for 100-seed weight among all the identified peak SNP (Figure 5C). The candidate genes of *Glyma.02G113600* (GO:0042631), *Glyma.02G115400* (GO:0006007), *Glyma.02G240400* (GO:0005986), *Glyma.04G131700* (GO:0010182), and *Glyma.04G228300* (GO:0005982) were selected as the strong candidate genes associated with 100-seed weight, which encode cellular response to water deprivation, glucose catabolic process, sucrose biosynthetic process, sugar mediated signaling pathway, and starch metabolic process, respectively (Figure 5C).

## 3. Discussion

Improving soybean seed composition traits has been an important criterion in most soybean breeding programs [62]. It has been well documented that soybean seeds contain a significant percentage of protein among all other legumes, with a range of 35–40% depending on the growing conditions and used cultivars [63]. However, the major impediment of developing high-seed protein soybeans is the negative correlation between yield and seed oil concentration. The linear Pearson correlation between soybean seed protein and seed oil was estimated to be −0.67 in this study. Soybean seed compositions are derived from glycolysis intermediates, which fuel the biosynthesis of protein and oil [64]. Glycolysis is known as the most important metabolic pathway that provides free energy by converting glucose into pyruvic acid to form the reduced nicotinamide adenine dinucleotide (NADH) and adenosine triphosphate (ATP) [65,66]. NADH and ATP are finally used to supply the required acetyl-CoA available for oil and protein synthesis [67]. Several studies reported that the seed oil and protein are synthesized at the seed development stage, and there is significant competition between these two traits in receiving acetyl-CoA [64,68]. Although simultaneous improvement in soybean seed protein and oil is still an important challenge in cultivar development programs, better identification of associated molecular markers with seed protein and oil is pivotal to breaking the existed negative correlations between both traits to some extent [3].

GWAS is currently considered as the most common way to discover MTAs for complex traits of interest [58]. However, more and more research is required to investigate the transferability and reproducibility of GWAS results across different genetic backgrounds and environments [69]. Several studies have reported inconsistent QTL identified for quantitative traits in different genetic backgrounds and across different environments. More specifically, several QTL have been found and reported for soybean seed protein, oil, and 100-seed weight [3,70], whereas a limited number of the detected QTL are currently used for marker-assisted selection in plant breeding programs due mainly to their inconsistent effects on the traits [69]. In general, there are several gaps in the use of the conventional GWAS methods for detecting MTAs for complex traits [71]. One of the major challenges with conventional statistical procedures is the “large p, small n” problem, which occurs when these methods are applied to datasets in which the number of markers is larger than the number of genotypes [16,69]. It is widely acknowledged that conventional GWAS methods are in general powerful for detecting common SNPs with large main effects that reach the level of significance [28]. Therefore, current conventional GWAS approaches are underpowered for discovering SNPs with minor effects underlying a given trait [72]. This study confirmed the efficiency of using an SVR-mediated machine learning algorithm in GWAS to detect reliable SNP markers associated with soybean seed composition traits. The use of SVR was investigated in predicting soybean yield and fresh biomass [73], wheat resistance [74], and in vitro breeding base methods [75]. The effectiveness of SVR in detecting more relevant MTAs for a trait of interest was demonstrated by de Oliveira et al. [38]. They compared different kernel types in SVR with other GWAS methods for detecting associated SNPs using simulation and real data in milk-related traits in cattle. The results showed that SVR had high potential to select associated SNPs markers for a trait of interest [38].

There are probable reasons why SVR was able to better detect the genomic regions associated with a trait of interest than FarmCPU. One of the important reasons is the ability of SVR to estimate significance levels for identifying SNP–trait associations using variable importance methods instead of the statistical methods used in conventional GWAS [71]. Variable importance allows for the consideration of interaction effects between SNPs, which is advantageous in identifying associations for complex traits. Conventional GWAS methods are better at detecting SNPs with large main effects on traits but are not as effective in considering the complex biological processes that shape these traits [23]. Recent studies have shown that SNPs with high importance scores may not necessarily have significant *p*-values from single SNP analyses [71,76,77]. Therefore, using variable importance values in SVR can improve the power of GWAS in discovering variant–trait associations with higher resolution [13].

In this study, SVR-mediated GWAS detected MTAs that were co-localized with two QTL directly related to soybean seed protein [48,55]. Most of the detected QTL for seed protein were also detected in separate environments. By selecting an appropriate GWAS method, the rate of detecting unstable MTAs will decrease, which can pave the way for using more MTAs in the MAS breeding strategy [78,79]. For soybean oil, most of the co-localized QTL with detected MTAs using SVR-mediated GWAS were related to the seed long-chain fatty acids reported previously by Fang et al. [55]. Seed long-chain fatty acids commonly contain 18–20 carbons, which can be categorized into different families based on the position of their first double-bound methyl end groups [80]. Triacylglycerols (TAGs), as important components of seed oil, are mostly composed of long-chain fatty acids [81]. Recent studies revealed the TAG biosynthesis pathway in soybean seeds [67,82,83]. Therefore, regulating the long-chain fatty acids may affect the overall seed oil percentage. In soybean 100-seed weight, SVR-mediated GWAS could find different MTAs co-localized with previously reported QTL related to this trait. 100-seed weight can be affected by several intrinsic and extrinsic factors, such as abiotic and biotic stresses, the total number of nodes and pods, and nutrition uptake [84,85]. Therefore, many genomic regions were involved in determining the ultimate 100-seed weight [84]. This study found that SCN and WUE related QTL in 100-seed weight, which shows the importance of biotic and biotic stresses in shaping this trait. It can be hypothesized that by improving the resistance to abiotic and biotic stress in new genotypes, a significant improvement in 100- seed weight can be achieved.

Several candidate genes related to seed protein (*Glyma.05G186700*, *Glyma.14G035100*, *Glyma.15G107800*, *Glyma.15G109300*, and *Glyma.19G068300*) were detected via SVR-mediated GWAS, which seems to have a direct influence in seed protein concentration. As an example, *Glyma.05G186700* encodes amino acid transport, which plays an important role in distributing essential nitrogen for plant growth and development [86]. *Glyma.14G035100* encodes tissue development which depends on the nitrogen distribution as encoded by *Glyma.05G186700*. *Glyma.15G107800* and *Glyma.15G109300* were other candidate genes for seed protein, which encode protein desumoylation and chloroplast organization, respectively. Those genes play important roles in maintaining energy production sites for supplying the required energy for storing seed compositions [87,88]. Three candidate genes (*Glyma.13G187100*, *Glyma.16G133500*, and *Glyma.16G133600*) were found to be strong candidate genes for soybean seed oil. *Glyma.13G187100* encodes methyltransferase activity, which plays a vital role in regulating tocopherols, an important component in the stability of soybean seed oil [89]. Another strong candidate gene for oil was *Glyma.16G133500*, which encodes salicylic acid biosynthetic process. Salicylic acid regulates the nitrate reductase activity in the plant, which plays an important role in increasing the protein and decreasing the oil percentages in seed [90,91]. Therefore, this gene may be useful in breaking the negative correlations between seed protein and oil percentage. From all the detected candidate genes for 100-seed weight, *Glyma.02G113600*, *Glyma.02G115400*, *Glyma.02G240400*, *Glyma.04G131700*, and *Glyma.04G228300* were selected as the strong candidate genes governing the trait. The candidate genes seem to be involved in glucose metabolism, specifically sugar catabolic processes. The breakdown of sugars is essential for providing energy during seed maturation and development. This gene may play a role in regulating the balance between glucose and other sugar molecules in the seed, contributing to the overall 100-seed weight. During the soybean seed maturity stages, the glucose level decreases significantly, while the levels of sucrose, sugar, and starch increase in the full mature soybean seed yield [92]. *Glyma.02G113600* encodes the glucose catabolic process responsible for breaking down the glucose to produce the primary sources of energy for the cellular production of ATP [93]. The produced ATP may be used in different biosynthesis (e.g., starch, sugar, and sucrose) and physiological processes [94,95].

Overall, the detected candidate genes for 100-seed weight are mostly involved in sugar, glucose, and starch metabolism. This suggests that the regulation of carbohydrate metabolism is crucial for determining seed size and weight. The breakdown of glucose and starch molecules provides the necessary energy and building blocks for seed development and growth. The balance between these different carbohydrate molecules is important for achieving optimal seed weight and size. Such information can be useful for soybean breeders to selectively breed for plants carrying favorable alleles of these candidate genes, resulting in soybean varieties with desired seed weight characteristics. Further research can be conducted to elucidate the precise roles of these genes in regulating carbohydrate metabolism and seed weight. This knowledge can contribute to a better understanding of plant physiology and can potentially be applied to other crops as well.

## 4. Materials and Methods

### 4.1. Plant Materials and Field Experiments

The GWAS panel, which consisted of 250 soybean genotypes, was grown in field conditions at the University of Guelph, Ridgetown campus in four environments (two locations × two years) in 2018–2019 at Ridgetown (42°27′14.8″ N 81°52′48.0″ W, 200 m above sea level) and Palmyra (42°25′50.1″ N 81°45′06.9″ W, 195 m above sea level), Ontario, Canada. The tested genotypes were derived from the core soybean germplasm, Ridgetown soybean breeding programs, that have been used for genetic studies and cultivar development activities. Field experiments were conducted using randomized complete block designs (RCBDs), with two replications in each tested environment. Each phenotypic plot consisted of five rows, which were 4.2 m long each, and the seeding rate was 50–57 seeds per m^2^. Nearest-neighbor analysis (NNA), as one of the well-known error control methods [96,97,98], was used to reduce the spatial variation and increase the accuracy of measured phenotypic data in each phenotypic plot.

### 4.2. Phenotypic Data and Analysis

Soybean seed yield (ton ha^−1^) was measured by harvesting three middle rows of each plot and adjusted based on days to maturity and 13% seed moisture. The total percentage of oil and protein in soybean seeds was measured via near-infrared reflectance (NIR) using a DA 7250 NIR analyzer (Perten Instruments Canada, Winnipeg, MB, Canada) on a dry weight basis. The used instrument was calibrated based on Perten Instruments [99,100]. Each NIR measurement was achieved by averaging three technical replicates. 100-seed weight was also measured based on adjusting to zero percent moisture (The raw phenotypic data is available at https://github.com/MohsenYN/Available-Datasets (accessed on 15 July 2023)). In order to estimate the average phenotype of the tested traits, the best linear unbiased prediction (BLUP) was used for each soybean genotype [101] using packages *lme4* [102] and AllInOne Pre-processing [103] in R software version 4.1.1. The possible outliers were detected using the proposed protocol by Bowley [98] and treated as missing data points. Overall, the following statistical model was used in this study (Equation (1)):

(1) $$Y=\mu +A_{x}+B_{z}+C_{i}+\varepsilon_{ij}\;$$

where *Y* stands for the trait of interest as a function of an intercept *μ*; *μ* is equal to the overall mean (fixed); *x* stands for the vector of block effects; *z* is the vector of the genotype effects (random), in which z ~ N(0, σ^2^_G_); *i* stands for the vector of random GxE interaction effects; and ε is equal to the vector of residuals, in which e ~ N(0, σ^2^_E_). *A*, *B*, and *C* represent the incidence matrices of *x*, *z*, and *i* effects, respectively.

In addition, the heritability (Equation (2)) of each tested trait was calculated based on the following equation:

(2) $$H^{2}=\frac{\sigma_{G}^{2}}{\sigma_{G}^{2}+\sigma_{E}^{2}}$$

where σ^2^_G_ is the genotypic variance and σ^2^_E_ stands for the environmental variance.

### 4.3. Genotyping

For extracting DNA, the collected trifoliate leaf tissues from the first rep of phenotyping plots at the Ridgetown location were freeze-dried for 72 h using a Savant ModulyoD Thermoquest (Savant Instruments, Holbrook, NY, USA). DNA of each soybean genotype was extracted using NucleoSpin Plant II kit (Macherey–Nagel, Düren, Germany), followed by a quality check through a Qubit^®^ 2.0 fluorometer (Invitrogen, Carlsbad, CA, USA). The genotyping-by-sequencing (GBS) step was performed using *ApeKI* [104] as one of the most common enzymatic digestions for soybean genotypes. Achieved SNPs were called from a total of 210 M single-end Ion Torrent reads using the Fast GBS pipeline [105], considering Gmax_275_v2 as the reference genome. The filtering process for SNPs was assessed using the (1) Markov model, (2) minor allele frequency less than 0.05, and (3) removing SNPs with more than 50% heterozygosity.

### 4.4. Analysis of Population Structure

The population structure analysis for the tested 227 soybean genotypes was conducted from a total of 17,958 high-quality SNPs using fastSTRUCTURE [106]. For this aim, five runs were performed for the number of population (K) from 1 to 15, and the optimum number of populations was selected via the K tool in fastSTRUCTURE software. Additionally, kinship was estimated and considered between genotypes to reduce the confounding in the tested GWAS population.

### 4.5. Association Analysis

Different GWAS methods may provide different results based on the population diversity, number of SNPs, and statistical power linked with each method [107]. Therefore, two different GWAS methods were tested to investigate their efficiency in detecting the most relevant MTAs for traits of interest. FarmCPU, as the most common GWAS method, divides the multi-locus mixed model into a random effect model (REM) and a fixed-effect model (FEM), then employs them iteratively to achieve the best results in a given dataset [108]. For setting a threshold in the FarmCPU method, the FDR was used properly [109]. A, rMVP package [110] in R software version 3.6.1 was used for all FarmCPU analyses. In general, FEM and REM equations are as follows:

(3) $$FEM(Y_{i})=C_{i1}D_{1}+C_{i2}D_{2}+C_{i3}D_{3}+\cdots +C_{it}D_{t}+M_{ij}K_{j}+e_{i}$$

(4) $$REM\left(Y_{i}\right)=U_{i}+e_{i}$$

where *Y_i_* represents the observation on the ith sample; *C*_*i*1_, *C*_*i*2_, …, *C_it_* is equal to the genotypes of the t pseudo-QTNs; *D*_1_, *D*_2_, *D*_3_, …, *D*_t_ stands for the corresponding effect for the pseudo-QTNs; *M_ij_* is equal to the genotype of the jth SNPs and ith sample; *K_j_* stands for the corresponding effect of the jth SNPs; *U_i_* is the total genetic effect of the ith sample; and *e_i_* is the residual.

SVR is based on creating a set of hyperplanes used in regression problems [32]. This algorithm was implemented in GWAS based on estimating the variable importance proposed by Weston et al. (2001) [111], where SNPs and traits of interest consider as input and output variables, respectively (Equation (5)):

(5) Y=Wβ(c)+b

where *Y* stands for the output, *W* is the weight for each high-dimensional input variable (β) which is considered non-linearly on the input space of (*c*). The lower and upper borderlines are created as Y=Wβ(c)+b−e and Y=Wβ(c)+b+e, respectively.

For SVR-mediated GWAS, the scaled method (0–100) was used for estimating the importance of each SNP associated with traits of interest. In order to implement the SVR method in GWAS, a five-fold cross-validation strategy with ten repetitions was applied to estimate the variable importance of each SNP [112]. Still, there is no confirmed way to set the significance threshold in the SVR-mediated GWAS. Therefore, the global empirical threshold [113,114] was used based on fitting the SVR algorithm, storing SNPs with the highest variable importance score, repeating the process 1000 times, and selecting the associated SNPs based on α = 0.5. SVR-mediated GWAS was conducted using the *Caret* package [115] in R software version 3.6.1.

### 4.6. Extracting Candidate Genes Undelaying Detected QTLs

**Figure 6:**
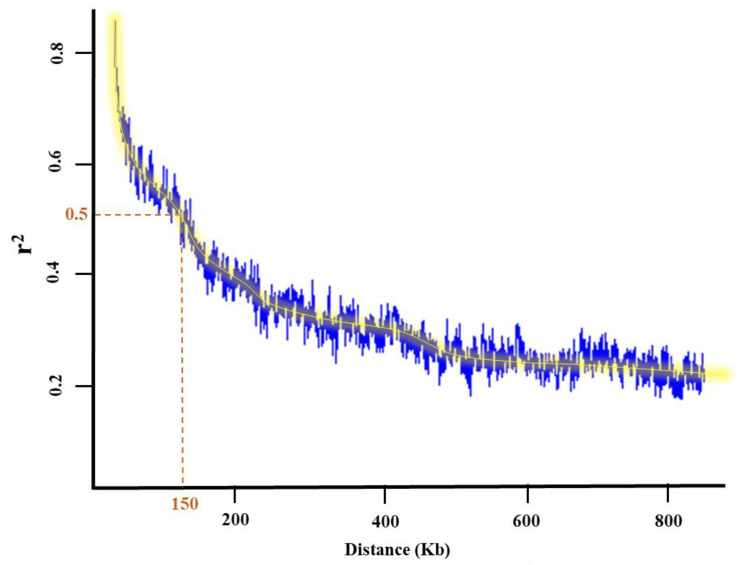
Linkage disequilibrium (LD) decay plot and the flanking regions depicting associated single nucleotide polymorphisms (SNPs) with traits of interest in a panel of 227 soybean genotypes.

One of the most common ways to verify QTL and candidate genes co-localized with MTAs detected using the tested GWAS methods is to investigate the functional annotation of candidate genes. The potential genes and QTL were retrieved based on the *G. max* William 82 reference gene models 2.0 in SoyBase (https://www.soybase.org (accessed on 15 July 2023)) on 150 k bp flanking regions of each MTA, identified using LD decay distance (Figure 6). Previous studies, gene ontology, and Go term enrichment (https://www.soybase.org (accessed on 15 July 2023)) were used as three criteria to detect the most relevant over-represented QTL and genes with a trait of interest.

## 5. Conclusions

This study suggests that improved GWAS methods, such as SVR-mediated GWAS, can enhance the understanding of the genetic basis of seed composition traits in soybeans. This understanding can be used by food-grade soybean breeders to develop more reliable and efficient cultivars. The study also identified a candidate gene, *Glyma.16G133500*, that could potentially break the negative correlation between seed oil and protein concentrations. Further investigation of the identified candidate genes and their differential gene expressions could lead to the development of gene-specific markers for marker-assisted selection (MAS) in soybean breeding programs. Overall, the results highlight the potential of advanced computational methods for improving the accuracy and efficiency of identifying MTAs and developing new soybean varieties with desired seed composition traits.

## Figures and Tables

**Table 1:** The list of MTAs for seed protein identified using FarmCPU and SVR-mediated GWAS methods in the combined environment dataset colocalized with reported QTL.

GWAS Method	Chromosome	MTA (Peak SNP Position)	Co-Located QTL	Environments ^a^	Reference
**FarmCPU**	S15	7068549	Shoot Fe 1-g43	NA	[39]
		7288161	SCN 5-g32	NA	[40]
		7705443	Seed protein 7-g13	NA	[41]
			Leaf carotenoid content 1-g11	NA	[42]
			WUE 2-g34	NA	[43]
		8304621	Shoot Zn 1-g24	NA	[39]
		8554284	Shoot Zn 1-g25	NA	[39]
		8620771	Shoot Zn 1-g26	NA	[39]
**SVR**	S01	50879523	Ureide content 1-g1.1	NA	[42]
		50933494	Ureide content 1-g1.2	NA	[42]
		50945345	Ureide content 1-g1.3	NA	[42]
		50947984	Ureide content 1-g1.4	NA	[42]
		51104169	First flower 2-g1	NA	[44]
		51797141	Canopy cover 1-g1	NA	[45]
		51104169	First flower 7-g1	NA	[44]
		51679239	Seed Trp 1-g1	NA	[46]
	S05	37483313	Shoot Mg 1-g4	2&4	[39]
		37414768	Shoot Cu 1-g6	2&4	[39]
		31380926	Seed oil 5-g1	2&4	[42]
		35536817	Pod number 3-g4	2&4	[47]
		31380926	Seed protein 4-g1	2&4	[48]
		37297357	Shoot Zn 1-g10.1	2&4	[39]
		37347763	Shoot Zn 1-g11	2&4	[39]
		37289637	Shoot P 1-g7	2&4	[39]
			Shoot Zn 1-g9	2&4	[39]
		37297357	Shoot P 1-g8.1	2&4	[39]
		37317508	Shoot P 1-g8.2	2&4	[39]
			Shoot Zn 1-g10.2	2&4	[39]
		37347763	Shoot P 1-g9	2&4	[39]
	S14	2919862	First flower 2-g20	NA	[44]
		3198128	Sclero 3-g56	NA	[49]
		3419976	Sclero 3-g57	NA	[49]
	S16	28851611	Seed protein 7-g25	1,2&4	[41]

^a^ Detected in separate environments in addition to the combined environment. (1) 2018Ridgetown, (2) 2019Ridgetwon, (3) 2018Palmyra, (4) 2019Palmyra, (NA) not found in any separate environment. FarmCPU: fixed and random model circulating probability unification, SVR: support vector regression.

**Table 2:** The list of MTAs for seed oil concentration identified by FarmCPU and SVR-mediated GWAS methods in the combined environment dataset colocalized with reported QTL.

GWAS Method	Chromosome	Peak SNP Position	Co-Located QTL	Environments ^a^	Reference
**FarmCPU**	S08	18259484	SDS 1-g54	NA	[50]
		18404800	SDS 1-g40	NA	[50]
			SDS 1-g55	NA	[50]
	S13	27301888	Shoot Fe 1-g33	NA	[39]
			SCN 1-g11	NA	[51]
		27325073	Shoot Fe 1-g34	NA	[39]
		33018554	SCN 4-g11	NA	[52]
	S15	7705443	Seed protein 7-g13	NA	[41]
			Leaf carotenoid content 1-g11	NA	[42]
			WUE 2-g34	NA	[43]
		8304621	Shoot Zn 1-g24	NA	[39]
		8554284	Shoot Zn 1-g25	NA	[39]
	S19	40386502	Iron deficiency chlorosis 4-g27	NA	[53]
		40550665	Iron deficiency chlorosis 2-g9	NA	[54]
			Iron deficiency chlorosis 3-g14	NA	[54]
**SVR**	S03	12702388	Seed long-chain fatty acid 1-g7.2	2	[55]
		12704607	Seed stearic 1-g2.2	2	[55]
		12917268	Seed long-chain fatty acid 1-g13.2	2	[55]
		12954110	Seed stearic 1-g2.3	2	[55]
		12958942	Seed long-chain fatty acid 1-g13.3	2	[55]
		12989558	Seed long-chain fatty acid 1-g7.3	2	[55]
	S13	30062400	Hilum color 2-g5.2	NA	[55]
			Hilum color 2-g5.3	NA	[55]
		30080662	Phytoph 3-g21	NA	[51]
		29941996	Soybean mosaic virus 1-g1	NA	[51]
		30037573	Salt tolerance 1-g9	NA	[56]
		30062400	Hilum color 2-g5.1	NA	[55]
	S14	3198128	Sclero 3-g56	3	[49]
		3419976	Sclero 3-g57	3	[49]
	S15	21479453	Iron deficiency chlorosis 4-g20	3	[53]
		49067066	WUE 1-g5	3	[57]
	S16	28851611	Seed protein 7-g25	3	[41]

^a^ Detected in separate environments in addition to the combined environment. (1) 2018Ridgetown, (2) 2019Ridgetwon, (3) 2018Palmyra, (4) 2019Palmyra, (NA) Not found in any separate environment. FarmCPU: Fixed and random model circulating probability unification, SVR: Support Vector Regression.

**Table 3:** The list of colocalized reported QTL with MTAs for 100-seed weight identified using FarmCPU and SVR GWAS methods in the combined environment dataset.

GWAS Method	Chromosome	Peak SNP Position	Co-Located QTL	Environments ^a^	Reference
**FarmCPU**	S18	703188	WUE 2-g47	NA	[43]
		713403	SCN 1-g16	NA	[51]
		822049	SCN 1-g17	NA	[59]
**SVR**	S02	11045403	Seed Trp 1-g5	2&4	[46]
		43004026	WUE 2-g7	2&4	[48]
	S03	38932768	Canopy width 1-g1.1	NA	[55]
		38936586	Canopy width 1-g1.2	NA	[55]
		39088673	R8 full maturity 3-g4	NA	[47]
	S09	42132672	Al tolerance 1-g9	NA	[60]
		42351295	Shoot K 1-g19	NA	[39]
	S11	4572326	SCN 5-g22	4	[40]
	S15	36329398	Ureide content 1-g42	NA	[42]
	S16	37330986	Seed linolenic 1-g10	2	[61]
		37153578	Shoot Cu 1-g15	2	[39]
		37330986	Seed palmitic 1-g14	2	[61]
			Seed oleic 1-g23	2	[61]
			Seed linoleic 1-g19	2	[61]
		37046875	WUE 2-g38	2	[40]
			Iron deficiency chlorosis 3-g10	2	[54]
		37079553	Node number 1-g5.1	2	[55]
		37079569	Node number 1-g5.2	2	[55]
		33018083	BSR 1-g2	2	[51]
	S19	47335622	Node number 1-g2.3	NA	[55]
	S20	276646	First flower 2-g25	1&2	[44]
			First flower 7-g25	1&2	[44]
		343016	Iron deficiency chlorosis 3-g15	1&2	[54]
		376574	Plant height 1-g26	1&2	[44]
			Plant height 6-g26	1&2	[44]

^a^ Detected in separate environments in addition to the combined environment. (1) 2018Ridgetown, (2) 2019Ridgetwon, (3) 2018Palmyra, (4) 2019Palmyra, (NA) not found in any separate environment. FarmCPU: fixed and random model circulating probability unification, SVR: support vector regression.

## Data Availability

All datasets will be freely available upon request.

## Supplementary Materials

The following supporting information can be downloaded at: https://www.mdpi.com/article/10.3390/plants12142659/s1, Table S1: The full list of detected MTAs for seed protein using FarmCPU in the tested soybean population. Table S2: The full list of detected MTAs for seed protein using SVR in the tested soybean population. Table S3: The full list of detected MTAs for seed oil using FarmCPU in the tested soybean population. Table S4: The full list of detected MTAs for seed oil using SVR in the tested soybean population. Table S5: The full list of detected MTAs for seed oil using FarmCPU in the tested soybean population. Table S6: The full list of detected MTAs for 100-seed weight using SVR in the tested soybean population.
