# Supplementary material for: Application of SVR-Mediated GWAS for Identification of Durable Genetic Regions Associated with Soybean Seed Quality Traits
Source: Plants (Basel). 2023 Jul 16;12(14):2659. doi: 10.3390/plants12142659 (PMC10383196; doi:10.3390/plants12142659)
Supplement: Supplementary file 1 [file plants-12-02659-s001.zip › plants-2421966-supplementary.pdf]

Table S1. The full list of detected MTAs for seed protein using FarmCPU in the tested soybean population.

Chromosome	SNP position	Effect	Standard error
3	15234716	-0.4359	0.11447
3	15234726	-0.4410	0.11548
15	7193889	0.6491	0.17382
15	7733816	0.7211	0.15725
15	7756557	0.6729	0.16671
15	7756572	0.6729	0.16671
15	7756641	0.7039	0.17017
15	7756662	0.7039	0.17017
15	7822658	0.5335	0.13837
15	7891365	0.7218	0.17185
15	7995655	0.6484	0.16515
15	8453911	0.7403	0.16148
15	8453979	0.6972	0.15861
15	8453988	0.8256	0.17338
15	8513793	0.7031	0.15797

FarmCPU: Fixed and random model circulating probability unification.

Table S2. The full list of detected MTAs for seed protein using SVR in the tested soybean population.

Chromosome	SNP position	Effect (Scaled)
1	50230454	71.92
1	50572171	74.33
1	50961340	74.90
1	51408405	80.65
1	51516560	76.34
1	51517034	75.62
1	51589860	83.10
1	51628579	74.62
1	51630871	75.98
1	51764985	72.28
1	51765084	73.54
1	51814345	78.86
1	51814474	84.16
5	31316535	80.73
5	35419945	88.20
5	35422388	90.50
5	37399766	71.80
6	47513336	74.57
12	33249488	86.41
12	33457196	86.41
12	33521414	87.10
14	3334156	73.81
14	3346285	85.68
14	9487231	92.58
15	5606405	75.33
16	28926313	74.98
16	8197092	75.84

SVR: Support Vector Regression.

Table S3. The full list of detected MTAs for seed oil using FarmCPU in the tested soybean population.

Chromosome	SNP position	Effect	Standard error
7	35743521	-0.2207	0.05870
8	18403233	-0.2627	0.06924
13	21486008	-0.4791	0.11468
13	27107431	0.2610	0.06902
13	27256136	0.2578	0.06893
13	32907711	0.2163	0.05782
15	7733816	-0.3556	0.09303
15	8453911	-0.3592	0.09563
15	8453988	-0.3933	0.10292
19	40498656	0.4538	0.11772

FarmCPU: Fixed and random model circulating probability unification.

Table S4. The full list of detected MTAs for seed oil using SVR in the tested soybean population.

Chromosome	SNP position	Effect (Scaled)
3	12843941	76.39
3	12882802	82.66
3	12967720	77.26
12	1816355	81.94
12	33249488	80.30
12	33457196	80.30
13	29958610	79.81
14	3346285	77.52
15	21518830	92.25
15	41559690	95.28
15	44633341	76.21
15	49106487	80.06
16	28926313	88.83

SVR: Support Vector Regression.

Table S5. The full list of detected MTAs for seed oil using FarmCPU in the tested soybean population.

Chromosome	SNP position	Effect	Standard error
10	10650787	0.4522	0.12225
10	10689110	0.4601	0.12322
18	812340	0.6335	0.14888

FarmCPU: Fixed and random model circulating probability unification.

Table S6. The full list of detected MTAs for 100 seed-weight using SVR in the tested soybean population.

Chromosome	SNP position	Effect (Scaled)
2	11159017	91.25
2	42949884	82.66
3	15293395	82.66
3	16429719	82.66
3	19258162	82.66
3	39075894	84.39
4	49895660	85.64
4	49954584	85.64
9	42241635	82.66
11	4712550	82.66
14	9782202	82.66
15	36291394	82.66
15	36291484	82.66
15	36454863	86.39
16	33116501	82.66
16	37051444	82.66
16	37187094	82.66
16	37306568	82.66
16	37375421	82.66
19	47350982	82.66
20	266113	82.66
20	26567270	82.66

SVR: Support Vector Regression.
